# A Female with Right-Sided Thoracic Kidney with Bochdalek Hernia: A Case Report

**DOI:** 10.31729/jnma.4747

**Published:** 2019-12-31

**Authors:** Sunil Kumar Daha, Anish Karn, Nikhil Shrestha, Neharika Shrestha, Sanjaya Paudyal, Niraj Giri

**Affiliations:** 1 Patan Academy of Health Sciences, Lalitpur, Nepal; 2 Oxford University Clinical Research Unit, Nepal, Lalitpur, Nepal

**Keywords:** *Bochdalek hernia*, *ectopic kidney*, *thoracic kidney*

## Abstract

The thoracic kidney is the rarest form of an ectopic kidney that usually present on the left thorax and twice more common in males. No case has been reported from Nepal and very few cases are reported worldwide. We report a 24 years-old female with right thoracic kidney with Bochdalek hernia diagnosed incidentally. We have included clinico-radiological and surgical findings of the case with a review of the literature.

## INTRODUCTION

The intrathoracic kidney is one of the rarer form of ectopic kidney.^1-3^ This usually present on the right thorax and twice as common in male than female.^1-4^ Usually, ectopic kidneys are asymptomatic and only a few cases present with either its complication or other respiratory or gastrointestinal conditions.^6^ Most of them are diagnosed incidentally and further confirmation requires advanced radiological investigations. Magnetic resonance urography and computed tomography (CT) is a radio-imaging modality of choice.^3^ In this article, we report a 24-years-old female with right intrathoracic kidney with Bochdalek hernia diagnosed incidentally by radiography while investigating for acute cholecystitis.

## CASE REPORT

A 24-year-old lady, who presented with complaint of abdominal pain over the right upper quadrant for seven months. The pain was insidious on the onset, gradually progressing, intermittent, pricking type, moderate to severe in intensity and non-radiating nature. It was aggravated by physical movement and the intake of foods. It usually relieved with analgesic intake. The severity of pain had been increased for the last 10 days. There was no history of nausea, vomiting, anorexia or weight loss. Bowel and bladder habit was usual. Past medical and surgical history was insignificant.

**Figure 1:**
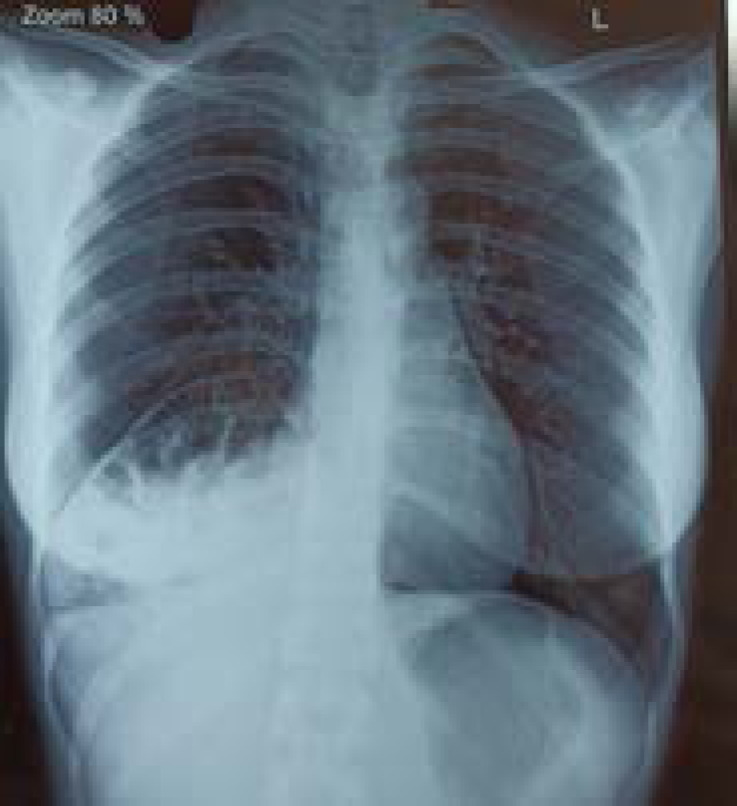
Chest x-ray showing eventration of diaphragm and bowel gases in the right thoracic region.

On examination, her general condition was fair and vitals were stable. Abdominal examination revealed tenderness over the right hypochondriac region and positive Murphy's sign. From history and examination, a provisional diagnosis of cholecystitis was made. Routine blood investigations, chest x-ray, ultrasonography (USG) of abdomen and pelvis were done. The USG findings were suggestive of calculus cholecystitis and right-sided ectopic kidney. The chest x-ray was suggestive of right-sided diaphragmatic hernia. All the routine investigations including renal function test were within normal limits (Figure 1).

**Figure 2:**
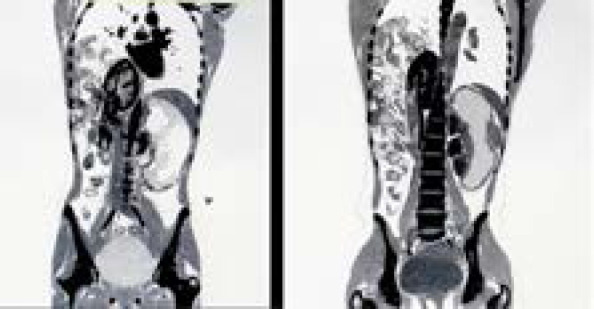
Left CT showing total eventration of right hemidiaphragm with segments of the bowel loop and right kidney in the thoracic cavity. Right CT showing functional right ectopic kidney.

**Figure 3:**
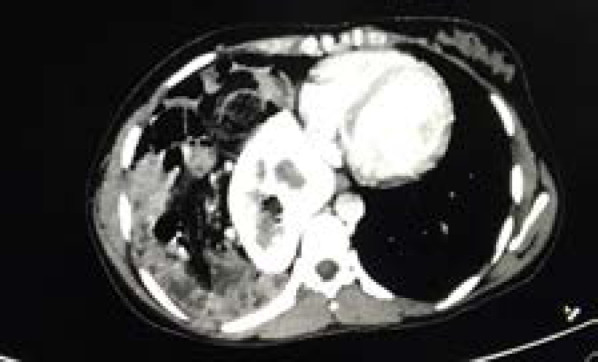
Axial view of CT showing the right kidney in the thoracic cavity near the pericardial region along with bowel loops.

CT abdomen was performed and showed total eventration of the right hemidiaphragm with superiorly displaced almost entire segments of the small bowel loops, hepatic flexure of the colon, associated mesentery and right kidney (Figure 2, 3). The right kidney lied at the thoracic region T-8 to the T-11 level with its arterial supply from the renal artery arising from the T-12 level from the abdominal aorta. The right renal artery showed early branching. The displaced right kidney was normal in size, outline, and attenuation. There was no focal lesion, calculus, hydronephrotic changes of the right kidney. There was intraluminal hyperdense calculus and biliary sludge was noted without wall thickening of the gall bladder. She was managed surgically with laparoscopic mesh repair of Bochdalek hernia with cholecystectomy. The intra-operative findings were a huge defect of the right posterolateral diaphragm (approximately 10 × 10 centimeters). Most of the small bowel and the colon was present in the thoracic cavity. The ectopic right kidney was lying over pericardium, high up in the thoracic cavity. There was a hypoplastic right lobe of the liver and hypertrophied left lobe of the liver. Post operative x-ray revealed no abnormality. She was kept in surgical intensive care (SICU) for observation and was discharged on 6^th^ post-operative day.

## DISCUSSION

Renal ectopy is a common congenital anomaly due to disrupted normal embryologic migration of the kidneys.^2^ The incidence of this anomaly is reported as 1 in 1000 autopsies. Renal ectopy result when the kidneys do not ascend to the retroperitoneal fossa. During the embryological period, by the end of 8 weeks of gestation, as the pleuroperitoneal membrane separates the pleural cavity from the peritoneal cavity, thus, the diaphragmatic leaflets are formed. Eventually, mesenchymal tissues associated with pleuroperitoneal membranes form the muscular components of the diaphragm. There is a significant decrease in the strength of the diaphragm muscle fiber. Diaphragm eventration has three anatomic forms: partial, often at right hemidiaphragm anteromedially; complete, which is usually localized at left hemidiaphragm; and bilateral.^7^

Their variants include (i) real thoracic ectopia with normally developed closed diaphragm, (ii) eventration of the diaphragm, (iii) diaphragmatic hernias [a) congenital diaphragmatic defects, b) acquired herniation (Bochdalek's)], and (iv) traumatic rupture of the diaphragm with renal ectopia.^5^ Thoracic renal ectopy is more common on the left side and affects males more than females.^1,8^

With renal ectopy patients, the majority of them are asymptomatic. The diagnosis is often made coincidentally during a routine radiographic investigation for either respiratory, gastrointestinal, renal, obstetrics or gynecology complaints.^2,8^ This anomaly has been found during prenatal ultrasonography and in all age groups.^4^ However, it has been mostly diagnosed in adults undergoing radiography for other indications.^6^ A case series that included 99 children with renal ectopy, of them 79 were asymptomatic. This diagnosis was made from antenatal or ultrasonography.^9^ Among patients diagnosed symptomatically, they usually present with abdominal pain, fever, hematuria or incontinence.^2^ Once detected, patients should be carefully looked for other anomalies. For other urological abnormalities, radiological testing including voiding cystourethrogram (VCUG), technetium 99m-mercaptotriglycylglycine (Tc99mMAG3) and 99mTc-diethylenetriamine penta-acetic acid (DTPA), etc. can be done. The prognostic outcome of these ectopic kidneys patients is excellent, provided if the absence of other anomalies. There is no clinical reports or autopsies series suggesting that a thoracic kidney will cause serious pulmonary or urologic complications.^3^

## Consent:

**JNMA Case Report Consent Form** was signed by the patient and the original is attached with the patient's chart.

## Conflict of Interest

**None.**
